# Comparison of Clinical Subtypes of Breast Cancer within the Claudin-Low Molecular Cluster Reveals Distinct Phenotypes

**DOI:** 10.3390/cancers15102689

**Published:** 2023-05-10

**Authors:** Ioannis A. Voutsadakis

**Affiliations:** 1 Algoma District Cancer Program, Sault Area Hospital, Sault Ste. Marie, ON P6B 0A8, Canada; ivoutsadakis@yahoo.com or ivoutsadakis@nosm.ca; 2 Division of Clinical Sciences, Northern Ontario School of Medicine, Sudbury, ON P3E 2C6, Canada

**Keywords:** genomic subtypes, clinical subtypes, epithelial mesenchymal transition, claudin-low, breast

## Abstract

**Simple Summary:**

Breast cancer is not one homogeneous disease, but it includes many subtypes that are identified in the clinical and research setting. The claudin-low subtype is a special subset of breast cancers that is associated with molecular alterations endowing cancer cells with a pro-metastatic phenotype. Claudin-low breast cancers represent up to two-fifths of breast cancers that are negative for the Estrogen Receptor and approximately 5% of cancers that are positive for the Estrogen Receptor. This article analyzes claudin-low cases of breast cancers contained in a published genomic cohort and compares them with cases that do not display the claudin-low type. It reveals significant differences in the prevalence of molecular alterations between claudin-low type cancers and cancers without this phenotype within the Estrogen Receptor negative and Estrogen Receptor-positive groups. These differences may have implications for targeted therapies and the design of clinical trials in breast cancer.

**Abstract:**

Background: Molecular subtyping of breast cancer has provided a new perspective on the pathogenesis of the disease and a foundation for building a clinical classification for this heterogeneous disease. The initial classification categorizing breast cancers into five groups, luminal A, luminal B, ERBB2-overexpressing, basal-like and normal-like, was later supplemented by an additional group, claudin-low tumors. However, the claudin-low group has been more difficult to align with clinically used immunohistochemical categories. The identity of this group among clinical cases remains ill defined. Methods: The METABRIC cohort comprising more than 1700 breast cancers and providing information for classifying them in both clinical groups and the genomic PAM50/claudin-low groups was analyzed to derive relationships and clarify potential pathogenic ramifications. Comparisons of the claudin-low cases bearing different clinical group classifications and of the respective cases with the same clinical non-claudin-low classifications were performed. Results: ER-negative/HER2-negative breast cancers are predominantly (88.4%) basal-like and claudin low. Conversely, most basal-like cancers (83.6%) are ER negative/HER2 negative. However, claudin-low breast cancers are only in 68.4% of cases ER negative/HER2 negative and the other clinical phenotypes, mostly ER positive/HER2 negative/low proliferation, are also represented in more than 30% of claudin-low cancers. These claudin-low non-ER-negative/HER2-negative breast cancers differ from claudin-low ER-negative/HER2-negative cases in grade, prevalence of integrative clusters, and prevalence of common mutations and common amplifications. Differences also exist between the two groups classified clinically as ER negative/HER2 negative, that are genomically basal-like or claudin-low, including in menopause status, grade, histology, prevalence of high tumor mutation burden, distribution of integrative clusters, prevalence of *TP53* mutations and of amplifications in the *MYC* and *MCL1* loci. Furthermore, distinct characteristics are observed between the luminal A and claudin-low groups within the clinical ER-positive/HER2-negative/low proliferation group. Conclusion: Within genomically claudin-low breast cancers, the ER-negative/HER2-negative group is distinct from the group with either ER or HER2 positivity. Conversely, within clinical phenotypes, claudin-low and non-claudin-low breast cancers differ in clinical characteristics and molecular attributes.

## 1. Introduction

The genomic classification of breast cancers based on gene expression derived from complementary DNA microarrays in the scheme proposed several years ago by Perou et al. categorizes breast cancers into five groups or intrinsic subtypes, luminal A, luminal B, ERBB2-overexpressing, basal-like and normal-like [1,2]. A surrogate classification used in the clinic takes advantage of the comparatively high concordance of the molecular subtypes and the protein expression of the Estrogen Receptor (ER), the Progesterone Receptor (PR) and the HER2 receptor (sometimes with the proliferation marker Ki67 as an add-on, referred to as the 3-gene classification) [3]. Luminal A cancers in the genomic classification are mostly ER positive/HER2 negative with low Ki67. Luminal B cancers in the genomic classification are usually also ER positive/HER2 negative but present high proliferation as shown by a high Ki67 in the 3-gene classification. ERBB2-overexpressing cancers in the genomic classification are HER2 positive with or without ER positivity in the clinical/immunohistochemical classification. The basal-like genomic subtype is mostly triple negative, staining negative for all three receptors, ER, PR and HER2. The normal-like genomic subtype is also often ER positive/HER2 negative, although heterogeneity is present. The concordance of genomic and clinical/immunohistochemical breast cancer subtypes is incomplete and several cases from each clinical group fall outside the most commonly corresponding genomic group. For example, in the three MONALEESA phase, three trials that compared hormonal therapy with or without ribociclib in ER-positive/HER2-negative breast cancers, only 70.7% of cases were luminal (46.7% luminal A and 24% luminal B) while 14% of cases were normal-like, 12.7% of cases were HER2 enriched and 2.6% of cases were basal-like [4]. Despite the imperfect concordance, the clinical classification has been used in clinics across the world for decades now and guides decision making both in adjuvant and advanced-stage breast cancers [5]. Based on the concept of genomic intrinsic subtypes, an abbreviated version of the classification derived from just 50 genes, called Prediction Analysis of Microarray 50 (PAM50), was developed for clinical use [6]. PAM50 can be performed in formalin-fixed paraffin-embedded samples using commercially available kits in a non-centralized manner.

Subsequent to the original intrinsic subtype classification, an additional genomic group of breast cancers was identified and named claudin-low due to down-regulation of adhesion proteins, such as claudin 3, 4 and 7, occludin and E cadherin [7]. This group had similarities with the basal-like group, displaying a low expression of luminal and HER2 clusters, but, in contrast to basal-like intrinsic subtype, it presented a low proliferation rate [8]. In addition, claudin-low tumors express clusters of genes shared with stem cells.

Triple-negative breast cancers, defined clinically as those breast cancers with absence of expression of ER, PR and HER2, are a heterogeneous subset, representing as a whole approximately 10% to 20% of breast cancers. Various genomic classification schemes have attempted to classify triple-negative cancers to different clusters based on expression profiles [9,10,11]. General themes that arise in this genomic triple-negative breast cancer subtyping include the identification of four rather stable categories [12]. A first separate category is a steroid receptor-dependent subtype that expresses the Androgen Receptor (AR), instead of ER and PR, which are both negative, by definition, at the protein level, as detected by immunohistochemistry. A basal-like immune-activated subtype is characterized by chromosomal instability, high expression of genes involved in interferon gamma (IFNγ) signaling and of genes encoding for checkpoint receptors, including PD-1 and CTLA4 and possess the common in triple-negative breast cancer mutations in *TP53*. The third subtype is also a basal-like subtype with high chromosomal instability and high *TP53* mutation rate, but in contrast to the basal-like immune-activated subtype, it is immune suppressed, displaying low expression of immune pathway genes and high expression of cell proliferation genes. The fourth genomic subtype of triple-negative breast cancer is called mesenchymal and is characterized by increased stromal, epithelial–mesenchymal transition and angiogenesis signatures as well as suppression of the immune presentation machinery [13]. Of interest, the mesenchymal subtype contains tumors that are classified into different groups of the PAM50 classification [12]. In addition, a proteomics analysis of breast cancers identifies three groups of breast cancers, two of them overlaying the luminal subtypes and the basal subtype of genomic classifications, and a third group dominated by stromal proteins expression and having a variable PAM50 classification assignment [14]. These data suggest that although the mesenchymal group is phenotypically triple-negative it includes cancers that fall into all breast cancer subgroups, as defined by genomic classifiers [15].

This investigation examines the characteristics of the subsets of claudin-low breast cancers according to their immunohistochemical classification in the METABRIC cohort and the relationship of the basal and claudin-low groups with the aim to pinpoint pertinent characteristics of clinical and prognostic significance within the clinically used subtypes. Hypotheses on the identity and pathogenesis of claudin-low cancers as an autonomous subtype or a mesenchymal transitioned state of other subtypes are also discussed.

## 2. Methods

The METABRIC (Molecular Taxonomy of Breast Cancer International Consortium) cohort of breast cancers as annotated in cBioportal for cancer genomics site was analyzed [16]. The primary data for the current study were extracted from the online cBioPortal for Cancer Genomics Portal (cBioportal, http://www.cbioportal.org, accessed on 14 March 2023). This online platform is a genomics site initially developed by Memorial Sloan Kettering Cancer Center (MSKCC) and currently maintained by MSKCC in collaboration with other academic institutions [17,18]. The platform is open access and user-friendly, as it requires minimal technical expertise for assessing and analyzing contained data. Interested investigators can interrogate the database of included genomic studies for any gene of interest examined in the original studies and for any molecular alteration, including mutations, copy number alterations and mRNA expression.

The METABRIC cohort is an extensive breast cancer genomic cohort that includes 2509 patients with 2173 patient available samples with copy number alterations (CNAs) for analysis. Among samples in METABRIC, 700 samples are of the luminal A subtype and 209 samples are basal. A pie graph tool in cBioportal allows selection of samples for the subtype and integrative cluster restricted analyses. Cases in METABRIC are assigned a 3-gene group classification, as well as a molecular classification according to the PAM50 plus claudin-low genomic classification and an integrative cluster classification. The integrative clusters classification is based on patterns of CNAs across the genome and assigns breast cancers into 11 clusters (clusters 1 to 10 with cluster 4 being subdivided into a 4ER+ and a 4ER- group) [19]. The 11 integrative clusters are characterized by variable patterns of chromosome segment alterations that include regional gains and losses. In addition, representational oligonucleotide microarrays are used to identify the most common chromosomes that present these altered patterns. The four main patterns revealed in the integrative cluster analysis, are a simplex pattern, two complex patterns, called complex I and complex II and a flat pattern. The simplex pattern presents an extensive distribution of broad amplified and deleted segments. The complex I pattern displays generalized narrow areas of duplications and deletions emulatng a sawtooth appearance. The complex II pattern possesses one or more localized areas of high level of amplifications intermixed with deletions called a firestorm pattern, and the fourth pattern displays no clear gains or losses of chromosomal regions. The simplex pattern observed in integrative clusters 7 and 8 is frequent in luminal A breast cancers. The complex I pattern is observed mostly in the integrative cluster group 10 which consists predominantly of basal-like breast cancers. The complex II pattern characterizes clusters containing luminal B and HER2-positive cancers.

Statistical comparisons of categorical and continuous variables were executed with the Fisher’s exact test or the x^2^ test and the *t* test, as appropriate. For survival analyses, Kaplan–Meier curves were constructed and were compared with the Log Rank test. All statistical comparisons were considered significant at a level of *p* < 0.05.

## 3. Results

The METABRIC study provided data on the clinical 3-gene classification category (ER positive/HER2 negative/low proliferation, ER positive/HER2 negative/high proliferation, HER2 positive, ER negative/HER2 negative) for most of the patients participating. The 3-gene classifier was not available in 745 patients of the cohort; and from the remaining 1764 patients, 5 patients could not be classified into a PAM50 or the claudin-low categories. Thus, a total of 1759 patients had both a molecular and clinical categorization (Table 1). The claudin-low subtype was present in 218 samples (8.7%) in the METABRIC cohort, 38 of which were not annotated for ER and HER2 status. From the remaining 190 samples, 130 (68.4%) were ER negative and HER2 negative (claudin-low/ER-negative/HER2-negative cohort), and 60 samples (31.6%) were ER positive or HER2 positive (claudin-low/ER-positive and/or HER2-positive cohort, Fisher’s exact test *p* = 0.0001 for the comparison of the percentage of claudin-low cases in ER-negative/HER2-negative cases and the percentage of claudin-low cases in ER-positive and/or HER2-positive cases). Most of these (39 of 60 samples, 65%) were ER positive/HER2 negative with low proliferation index, 12 samples (20%) were HER2 positive and 9 samples (15%) were ER positive/HER2 negative with high proliferation. ER-negative and HER2-negative samples with a basal-like genomic subtype (basal-like/ER-negative/HER2-negative cohort) were observed in 143 samples (8.1% of the 1764 samples with available data for determination of the clinical 3-gene classifier). A comparison of the menopause status at presentation between the two ER-negative/HER2-negative groups showed that the basal-like/ER-negative/HER2-negative cohort had a pre-menopausal prevalence that was statistically significantly higher than the claudin-low/ER-negative/HER2-negative cohort (Fisher’s exact test *p* = 0.01, Table 2). The two claudin-low cohorts did not differ significantly in the percentage of pre-menopausal women at diagnosis (Fisher’s exact test *p* = 0.15), although the claudin-low/ER-negative/HER2-negative cohort had a higher percentage of pre-menopausal women, which was intermediate between the two other groups (Table 2). The claudin-low/ER-positive and/or HER2-positive cohort had a higher prevalence of lobular cancers than the two ER-negative/HER2-negative cohorts, reflecting the association of this histology with ER positivity [20]. In contrast, the claudin-low/ER-negative/HER2-negative cohort had 12.9% of cases with non-ductal or lobular histology. These included 10 cases (8.1%) with medullary histology. The basal ER-negative/HER2-negative cohort had a higher percentage of cases with high cellularity, while the two claudin-low groups showed no significant differences in cellularity (Fisher’s exact test *p* = 0.4).

Regarding tumor mutation burden (TMB), both claudin-low groups had very low numbers of cases with a TMB above 10 mutations/Mb, while the basal ER-negative/HER2-negative group had a high TMB in 14% of cases (Table 3). The dominant integrative cluster in claudin-low tumors was the integrative cluster 4ER+ in the claudin-low/ER-positive and/or HER2-positive cohort, while the claudin-low/ER-negative/HER2-negative cohort cases were divided between integrative cluster 4ER+ and 4ER-. The basal ER-negative/HER2-negative group possessed only few cases of clusters 4ER+ and 4ER-. The majority of cases in the basal ER-negative/HER2-negative group belonged to integrative cluster 10. Integrative cluster 10 was also present but had a lower prevalence in the claudin-low/ER-negative/HER2-negative group, while no cases of the claudin-low/ER-positive and/or HER2-positive group belonged to this integrative cluster (Table 3).

**Figure 1:**
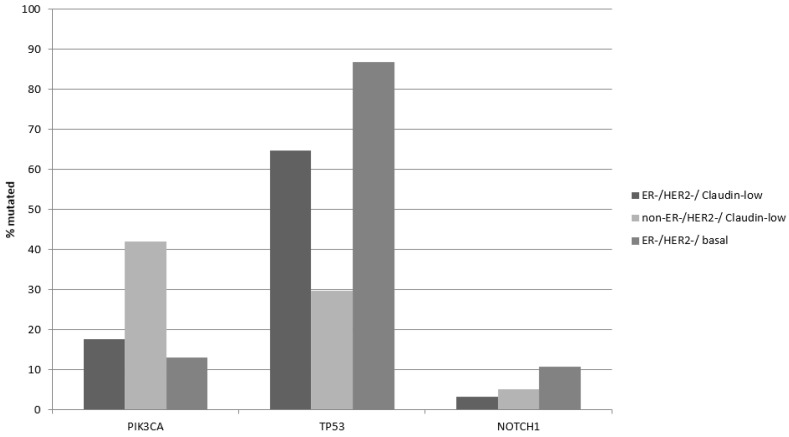
Prevalence of mutations in the *PIK3CA*, *TP53* and *NOTCH1* genes in ER-negative/HER2-negative claudin-low breast cancers, non-ER-negative/HER2-negative claudin-low breast cancers, and ER-negative/HER2-negative basal breast cancers. Statistical comparisons for each of the three genes presented are shown in Table 4.

The most prevalent oncogene mutated in breast cancer is the gene encoding for the catalytic alpha subunit of kinase PI3K, *PIK3CA*. Mutations in *PIK3CA* were more prevalent in claudin-low/ER-positive and/or HER2-positive breast cancers in the METABRIC cohort (42.1%) compared with claudin-low/ER negative/HER2 negative (17.6%, Fisher’s exact test *p* = 0.0001, Table 4, Figure 1), reflecting the higher prevalence of these mutations in ER-positive disease. In contrast, the two ER-negative/HER2-negative groups had similar prevalence of *PIK3CA* mutations (Figure 1). Mutations in the gene encoding for tumor suppressor p53, *TP53* were more prevalent in claudin-low/ER negative/HER2 negative (64.7%) compared with 29.8% in claudin-low/non-ER negative/HER2 negative (Fisher’s exact test *p* = 0.0001, Table 4). On the contrary, claudin-low/ER-negative/HER2-negative cancers had a lower prevalence of *TP53* mutations than basal ER-negative/HER2-negative cancers, which showed TP53 mutations in 86.9% of cases (Fisher’s exact test *p* = 0.0001, Table 4). The higher prevalence of *TP53* mutations reflects their predominance in triple-negative breast cancer compared with ER-positive disease. Oncogene *NOTCH1* displayed also higher mutation rates in basal ER-negative/HER2-negative cancers, being the third most frequently mutated gene in this group (Figure 1).

Regarding copy number alterations, the amplification of locus 8q24.21, where oncogene *MYC* and stemness factor *POU5F1B* reside, was similarly present in the two claudin-low groups, but more prevalent in basal ER-negative/HER2-negative cancers (Table 5). This group had also a higher prevalence than the two claudin-low groups of amplification in loci of chromosome 1q, where the genes *MCL1* and *NTRK1* reside. The claudin-low/non-ER-negative/HER2-negative group had a higher prevalence than claudin-low/ER-negative/HER2-negative cancers of amplifications in loci 11q13.3, 17q12 and 8p11.23, encoding for the genes *CCND1*, *ERBB2* and *NSD3*, respectively (Table 5).

**Figure 2:**
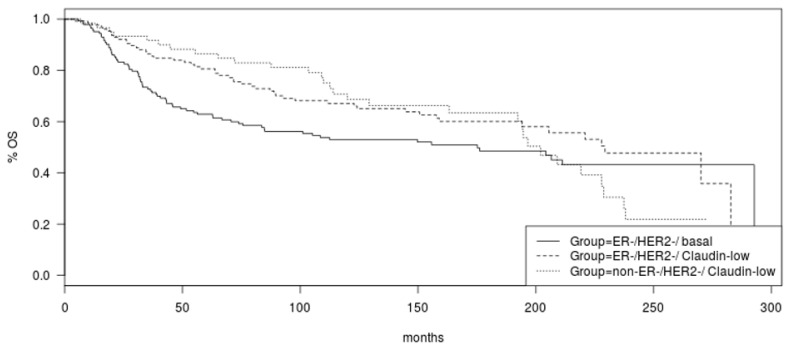
Overall survival of the groups of ER-negative/HER2-negative claudin-low breast cancers, non-ER-negative/HER2-negative claudin-low breast cancers, and ER-negative/HER2-negative basal breast cancers. Log Rank *p* = 0.53 for the comparison between the two claudin-low groups and Log Rank *p* = 0.06 for the comparison between the two ER-negative/HER2-negative groups.

Overall survival (OS) did not differ significantly between the two claudin-low groups, ER negative/HER2 negative and non-ER negative/HER2 negative (Log Rank *p* = 0.53, Figure 2). A trend towards better OS of the ER-negative/HER2-negative/claudin-low group compared to basal-like counterparts was observed but did not reach significance (Log Rank *p* = 0.06, Figure 2).

The comparison of the ER-positive/HER2-negative/low proliferation/claudin-low group (*n* = 39) with the ER-positive/HER2-negative/low proliferation/luminal A group (*n* = 471) showed no significant differences in menopause status, grade and histology of the tumors (Table 6). The claudin-low group contained more samples with low cellularity.

The TMB was low in most cases in both ER-positive/HER2-negative/low proliferation groups (Table 7). The luminal A group had 6.2% of cases with high TMB (above 10 mutations/Mb), while the claudin-low group had no cases with high TMB. Most cases of the luminal A group belonged to integrative clusters 3 and 8, while the majority of cases in the ER-positive/HER2-negative/low proliferation/claudin-low group belonged to integrative cluster 4ER+ (Table 7).

**Figure 3:**
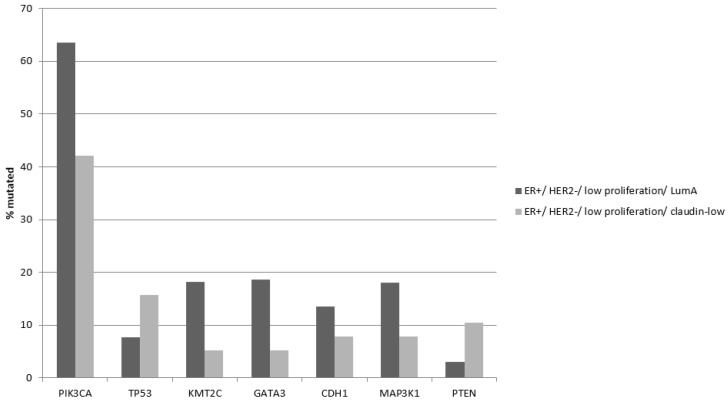
Prevalence of mutations in the most frequently breast cancer mutated genes in ER-positive/HER2-negative/low proliferation/claudin-low breast cancers and ER-positive/HER2-negative/low proliferation/luminal A breast cancers. Statistical comparisons for each gene presented are shown in Table 8.

Common breast cancer mutations in the *PIK3CA*, *GATA3* and *KMT2C* genes are significantly more prevalent in ER-positive/HER2-negative/low proliferation/luminal A cancers compared to the claudin-low group (Table 8). In contrast mutations in *TP53* are more prevalent in the claudin-low group, although the difference did not reach statistical significance and their prevalence (15.8%) is lower than in both basal and claudin-low ER-negative/HER2-negative cancers (86.9% and 29.8%, respectively, Table 4 and Table 8). Tumor suppressor PTEN was also more frequently mutated in the claudin-low group of ER-positive/HER2-negative/low proliferation cancers (10.5% versus 3.1% in luminal A cancers, Fisher’s exact test *p* = 0.04, Figure 3).

Amplifications of MCL1 (1q21.2) and NTRK1 (1q23.1) are more prevalent in ER-positive/HER2-negative/low proliferation/luminal A cancers than in ER-positive/HER2-negative/low proliferation/claudin-low cancers (Fisher’s exact test *p* = 0.0001 and 0.001, respectively, Table 9), while other commonly amplified loci in breast cancer do not show significant differences in the two groups, despite being numerically more prevalent in claudin-low cases.

**Figure 4:**
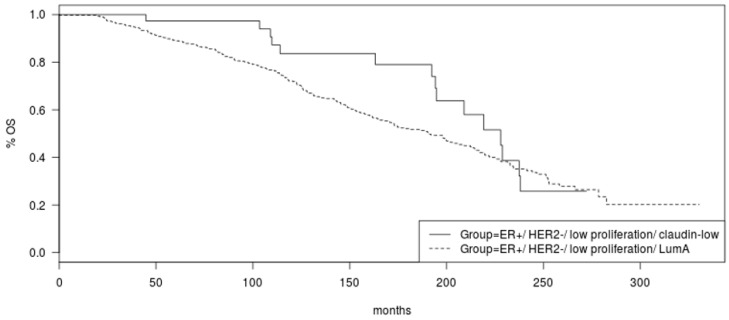
Overall survival of the groups of ER-positive/HER2-negative/low proliferation/luminal A and ER-positive/HER2-negative/low proliferation/claudin-low breast cancers (Log Rank *p* = 0.16).

OS of the two groups of ER-positive/HER2-negative/low proliferation breast cancers was not statistically different (Log Rank *p* = 0.16, Figure 4). Inspection of the survival curves reveals that there is a trend towards better OS for the claudin-low group (Figure 4).

## 4. Discussion

EMT is a process during embryo development that provides the developing organism with the tools for cell movement and organogenesis [21]. In adult organisms, EMT is involved in wound healing through mobilization of cells necessary for regeneration of injured tissue [22]. Beyond its physiologic roles, EMT of cancer is usurped by transformed cells enabling their ability to invade through tissue and metastasize [23,24]. Cells of epithelial cancers undergoing EMT lose their connections with neighboring cells through down-regulation of adhesion molecules, such E cadherin, occludin and ZO-1, and acquire mesenchymal features [25]. EMT is guided by a set of core transcription regulators that include ZEB1, ZEB2, Snail and Slug [26]. The transition of an epithelial cell into acquiring mesenchymal features is gradual and intermediate states exist. In addition, reversal of the process takes also place, triggered by environmental cues, and is termed mesenchymal to epithelial transition (MET). Thus, EMT and MET in cancer represent a fluid state that is sometimes referred to as Epithelial to Mesenchymal Plasticity (EMP). Plasticity is also a characteristic of stem cells and cancer stem cells [27]. Indeed, the close association of EMT with the stemness state has also been described and promotes the survival of metastatic cells and of cells with metastatic potential [28,29]. In breast cancer, transcriptional programs associated with stemness core transcription factors, such as Oct4, Nanog and Sox2, promote EMP and, reciprocally, EMT transcription regulators influence stem cell characteristics [29,30]. Moreover, both EMT and stemness are regulators of ER and other steroid receptors and are regulated by ER and PR [31,32].

The claudin-low group of breast cancers has molecular characteristics suggesting associations with the EMT process [33]. As the name implies, cells with the claudin-low phenotype display a low expression of tight junction adhesion proteins claudins 3, 4 and 7, as well as other adhesion proteins such as occludin and E cadherin. Despite E cadherin’s down-regulation, claudin-low cancers do not display lobular histology, implying that concomitant down-regulations or other molecular lesions obliterate development of lobular morphology and favor ductal features. In addition, E cadherin down-regulation in claudin-low cancers, in contrast to lobular cancers, is not due to genetic lesions in its gene but is due to epigenetic or post-transcriptional deregulation. Moreover, claudin-low cancers display low expression of epithelial surface molecules CD24 and EpCAM and high expression of CD44 and CD49f [33]. This profile parallels the profile of mammary stem cells [34]. In addition, claudin-low tumors show a higher percentage than other subtypes of cells with dual positivity for epithelial markers, such as cytokeratins 5 and 19, and for mesenchymal markers, such as vimentin [33].

In the current analysis from the METABRIC dataset, claudin-low breast cancers are shown to vary within their group, depending on the clinical phenotype (ER and/or HER2 positive versus ER negative/HER2 negative). ER-positive and/or HER2-positive/claudin-low breast cancers represent approximately one-third of all claudin-low cancers and thus, attempts to use triple negativity in immunohistochemistry (potentially together with other markers) as a surrogate for claudin-low status would miss this significant subset [35]. ER-positive and/or HER2-positive/claudin-low breast cancers are more commonly low grade, lobular and belong to integrative cluster 4ER+ than ER-negative/HER2-negative/claudin-low counterparts. Mutations in *PIK3CA* and *TP53* predominate according to the underlying phenotype (*PIK3CA* mutations are more common in the ER-positive and/or HER2-positive group and *TP53* mutations are more frequent in the ER-negative/HER2-negative group). Conversely, in both clinical phenotypes with significant percentages of genomically claudin-low cancers, the ER-positive/HER2-negative/low proliferation phenotype and the ER-negative/HER2-negative phenotype, claudin-low cancers differ in several characteristics and molecular attributes compared with the dominant cancers in the phenotype, luminal A and basal-like, respectively. Interestingly, claudin-low cancer groups, independently of the clinical phenotype, had a higher percentage of cases with low cellularity than non-claudin-low cancers, emphasizing the idea that the attributes of claudin-low cancers may be in part due to a significant stromal element [15,35]. Prognosis of claudin-low cancers was not statistically different from non-claudin-low cancers of the same clinical phenotype, but a trend towards better survival of claudin-low cancers existed in both cases. In contrast, within the claudin-low group the underlying clinical phenotype had no bearing in survival. This suggests that low proliferation which is a characteristic of claudin-low breast cancers overshadows other molecular programs associated with ER and HER2 levels of expression.

The heterogeneity of claudin-low tumors has been suggested in a report that proposed three distinct subsets of claudin-low breast cancers, related to luminal, basal-like and normal mammary stem cells, respectively [36]. Each claudin-low subset segregated into different integrative clusters, with the stem cell subset (termed CL1) segregating exclusively to integrative clusters 4ER+ and 4ER-, the luminal subset (termed CL2) segregating mostly into luminal-related clusters 3 and 6 and one-third into the 2 clusters 4 (4ER+ and 4ER-), and the basal-like subset (termed CL3) segregating in equal parts between luminal clusters, the 2 clusters 4 and the basal-related cluster 10 [36]. The three claudin-low subsets did not completely overlap with ER/PR and HER2 phenotypes, as CL2 contained 18% of triple-negative cases and CL3 and CL1 contained 41% and 22% of ER/PR-positive cases, respectively.

The idea of heterogeneity of claudin-low breast cancers is also discussed in a report by Fougner et al. that analyzes claudin-low cases of the METABRIC cohort in relationship to their intrinsic genomic subtype [15]. This work compares claudin-low breast cancers with non-claudin-low cancers of the underlying genomic intrinsic subtype from which they have been derived in each case, rather than with the immunohistochemical phenotype as presented in the current report. As a result of the different comparator categories and methods used to affirm the claudin-low phenotype, the groups produced differ in numbers in the two reports. For example, a significant subset of claudin-low cancers in the Fougner et al. study belongs to a normal-like claudin-low category which does not exist in the current report, as normal-like tumors are not part of the immunohistochemical categorization. Despite differences in group construction, the two reports agree that the claudin-low breast cancer subsets are in several molecular characteristics more similar to their respective genomic and immunohistochemical/clinical groups than between them. Moreover, the survival outcomes of the claudin-low cancer patients within each genomic group are similar to the survival of non-claudin counterparts. The integration of claudin-low cancers with the immunohistochemical categories used in clinical practice is potentially more relevant for the wider clinical application of the findings of the current study in identifying claudin-low patients and tailor their treatment accordingly, should effective targeted therapies for claudin-low disease become available.

A universal feature of all claudin-low groups is low genomic instability. Claudin-low cancers have rarely a high TMB. In the METABRIC cohort only 2 cases had more than 10 mutations/Mb. Similarly, chromosomal instability (CIN) is low in general, although some heterogeneity exists. The above discussed subsets of claudin-low cancers illustrates this heterogeneity with CL1 cases having the lowest CIN as measured by the Fraction Genome Altered (FGA), CL2 cases having an intermediate mean FGA, similar to luminal A cancers and the CL3 group having the highest mean FGA of the three groups, similar to basal-like cancers [36]. Low CIN in claudin-low cancers may relate to the activity of EMT master transcription factor ZEB1, which protects from oncogene induced DNA damage and the resulting response associated with activation of p53 [37]. Thus, cells with EMP and stemness features may be resistant to treatments that rely on induction of DNA damage response.

In a model of induced loss of p53 in mammary luminal epithelial cells in mice in vivo, mammary tumors that develop after long-term suppression of p53 display claudin-low molecular characteristics with suppressed expression of claudins 3, 4 and 7 and up-regulation of the EMT genes *zeb1* and *twist1* and mesenchymal markers N cadherin and vimentin [38]. Thus, claudin-low cancers result from luminal cells through loss of p53 under these experimental conditions, suggesting that diverse cells may give rise to cells with EMT characteristics, with accumulation of molecular defects. The presence of p53 mutations in luminal A or in basal-like breast cancers was not prognostic for breast cancer specific survival in the METABRIC cohort [39]. Oncogene *KRAS* activation by endogenous mutations or exogenous expression in luminal epithelial breast cells also promotes development of neoplastic lesions that evolve to claudin-low or basal-like cancers with triple-negative phenotypes, in a mouse model [40]. Derived claudin-low cancers, in this model, present lower Ki67 proliferation marker expression and higher immune cell infiltrates than basal-like cancers, similarly to corresponding human disease. Persisting activity of KRAS is required for the maintenance of expression of EMT-related proteins and stemness markers. In another mouse model of HER2-positive mammary cancer, HER2 loss led to development of cancers with claudin-low features [41]. After loss of HER2, cells from these tumors growing in vitro acquired a spindle-like phenotype and a gradual increase in cells with the stem cell signature CD44^high^/CD24^negative^ [41]. Transfection of luminal MCF-7 breast cancer cells with the core transcription factors Snail or Slug induced features of claudin-low phenotype, including down-regulation of ER, epithelial cytokeratins and claudins [42]. In addition, up-regulation of genes of the TGF-β pathway was observed after Snail and Slug expression. Conversely, miR-200 family microRNAs are expressed in low levels in claudin-low breast cancer cells and their up-regulation alters the phenotype of claudin-low cell lines to a more epithelial morphology and reduces proliferation and metastasis [43]. Although core EMT proteins were suppressed in variable degrees after miR-200 microRNAs expression, an association with the program regulated by the Polycomb Repressive Complex 2 (PRC2) protein SUZ12 was observed in this model. Consistently, trimethylation of histone 3 at lysine 27 (H3K27me3), the target enzymatic activity of PRC2 was increased in cells expressing miR-200 microRNAs. Epigenetic regulations provide an opportunity of altering cell states between epithelial and mesenchymal phenotypes as part of the EMP continuum with less extensive underlying alterations of the genome itself. Different states across the EMP spectrum are operative during the different stages of breast carcinogenesis and metastasis establishment [44]. An additional layer of complexity is provided by the fact that signaling cascades influencing EMP may promote the epithelial or the mesenchymal phenotype depending on concomitant signaling cues. For example, interferon signaling is up-regulated in claudin-low breast cancer cells and is required for TGF-β mediated EMT, but it is also required for the maintenance of the epithelial identity in mammary cell not undergoing EMT [45].

The low proliferation rate of claudin-low phenotype of breast cancers is consistent with cells that possess stem cell characteristics. These cells are resistant to therapies and as a result may respond less favorably to different treatments compared to breast cancers of similar phenotype but without claudin-low features [46]. Taking into account the presence of claudin-low phenotype in breast cancers of various subtypes could promote prognostication and the ability to predict response to therapies and may help develop targeted treatments based on vulnerabilities associated with the claudin-low phenotype.

## Figures and Tables

**Table 1:** Clinical groups classified according to ER, HER2 and proliferation index (3-gene classifier) and corresponding genomic PAM50/claudin-low groups in the METABRIC cohort. Percentages represent cases according to the genomic classification within the clinical groups.

PAM50/Claudin-Low Groups	ER+/HER2-/Low Proliferation		ER+/HER2-/High Proliferation		HER2+		ER-/HER2-	
	*n*	%	*n*	%	*n*	%	*n*	%
Luminal A	471	73.7	162	26.4	16	8	0	
Luminal B	32	5	357	58.3	34	17.2	1	0.3
HER2+	10	1.6	53	8.6	106	53.5	24	7.8
Basal	0		10	1.6	18	9.1	143	46.3
Claudin-low	39	6.1	9	1.5	12	6.1	130	42.1
Normal-like	87	13.6	22	3.6	12	6.1	11	3.6
Total	640	100	617	100	198	100	309	100

**Table 2:** Clinical and pathologic characteristics of ER-negative/HER2-negative claudin-low breast cancers, claudin-low breast cancers with ER and/or HER2 positivity and of basal breast cancers. Data are from METABRIC. Two statistical comparisons are presented for each characteristic, the first is between the two claudin-low groups and the second is between the two ER-negative/HER2-negative groups. The Fisher’s exact test has been used for all comparisons besides comparisons of histologic types where the x^2^ test was used. NA: not available.

	Claudin-Low				Basal			
Characteristic	ER-/HER2- (*n* = 130)	%	ER+ and/or HER2+ (*n* = 60)	%	ER-/HER2- Basal (=143)	%	*p* for Comparison between the 2 Claudin-Low Groups	*p* for Comparison between the 2 ER-/HER2- Groups
Menopause status								
Pre-	38	29.2	11	18.3	62	43.4	0.15	0.01
Post-	92	70.8	49	81.7	81	56.6		
Grade								
I/II	27	22	30	53.6	14	9.9	0.0001	0.01
III	96	78	26	46.4	128	90.1		
NA	7		4		1			
Histology								
Ductal	95	76.6	41	71.9	130	93.6	0.02	0.0004
Lobular	4	3.2	8	14	4	2.9		
Mixed	9	7.3	5	8.8	2	1.4		
Other	16	12.9	3	5.3	3	2.1		
NA	6		3		4			
Specimen cellularity								
Low	30	24.6	13	23.6	11	7.9	0.4	0.0001
Intermediate	48	39.3	27	49.1	36	25.7		
High	44	36.1	15	27.3	93	66.4		
NA	8		5		3			

**Table 3:** TMB and integrative clusters in ER-negative/HER2-negative claudin-low breast cancers, claudin-low breast cancers with ER and/or HER2 positivity and of basal breast cancers. Data are from METABRIC. Two statistical comparisons are presented for TMB and for the most prevalent integrative clusters, 4ER+ and 4ER-, the first is between the two claudin-low groups and the second is between the two ER-negative/HER2-negative groups. For the prevalent in ER-negative/HER2-negative groups integrative clusters 10 only comparison between these group is presented as the claudin-low group with ER and/or HER2 positivity has no cases with this cluster. The Fisher’s exact test has been used for all comparisons. NA: not available; TMB: tumor mutation burden; IntClust: integrative cluster.

	Claudin-Low				Basal			
Characteristic	ER-/HER2- (*n* = 130)	%	ER+ and/or HER2+ (*n* = 60)	%	ER-/HER2- Basal (=143)	%	*p* for Comparison between the 2 Claudin-Low Groups	*p* for Comparison between the 2 ER-/HER2- Groups
TMB								
≤10 mutations/Mb	107	99.1	50	98	117	86	0.54	0.0001
>10 mutations/Mb	1	0.9	1	2	19	14		
NA	22		9		7			
Integrative Cluster								
IntClust 1	1	0.8	2	3.3	4	2.8		
IntClust 2	1	0.8	2	3.3	1	0.7		
IntClust 3	2	1.5	7	11.7	1	0.7		
IntClust 4ER+	35	26.9	34	56.7	5	3.5	0.0002	0.0001
IntClust 4ER-	33	25.4	2	3.3	7	4.9	0.0001	0.0001
IntClust 5	1	0.8	8	13.3	0			
IntClust 6	1	0.8	1	1.7	0			
IntClust 7	0		3	5	1	0.7		
IntClust 8	0		0		1	0.7		
IntClust 9	3	2.3	1	1.7	12	8.4		
IntClust 10	53	40.8	0		111	77.6	-	0.0001

**Table 4:** Prevalence of most common mutations in ER-negative/HER2-negative claudin-low breast cancers, claudin-low breast cancers with ER and/or HER2 positivity and of basal breast cancers. Data are from METABRIC. Two statistical comparisons are presented for each gene (except for cases with no mutations), the first is between the two claudin-low groups and the second is between the two ER-negative/HER2-negative groups. The Fisher’s exact test has been used for all comparisons.

	Claudin-Low				Basal			
Gene	ER-/HER2- (*n* = 119)	%	Non-ER-/HER2- (*n* = 57)	%	ER-/HER2- Basal (=137)	%	*p* for Comparison between the 2 Claudin-Low Groups	*p* for Comparison between the 2 ER-/HER2- Groups
PIK3CA	21	17.6	24	42.1	18	13.1	0.0008	0.38
TP53	77	64.7	17	29.8	119	86.9	0.0001	0.0001
KMT2C	9	7.6	5	8.8	10	7.3	0.77	0.99
GATA3	2	1.7	2	3.5	0		0.59	-
CDH1	2	1.7	3	5.3	2	1.5	0.08	1.0
MAP3K1	2	1.7	4	7	4	2.9	0.08	0.68
KMT2D	9	7.6	0		10	7.3	-	1.0
PDE4DIP	9	7.6	5	8.8	13	9.5	0.77	0.65
TBX3	1	0.8	4	7	1	0.7	0.03	1.0
NOTCH1	4	3.4	3	5.3	15	10.9	0.68	0.03
ARID1A	3	2.5	2	3.5	6	4.4	0.65	0.51
CBFB	0		4	7	0		-	-
NCOR2	8	6.7	3	5.3	6	4.4	1.0	0.42
AKT1	1	0.8	2	3.5	3	2.2	0.24	0.62
PTEN	6	5	4	7	10	7.3	0.72	0.60
RB1	5	4.2	0		8	5.8	-	0.58
ROS1	6	5	1	1.8	10	7.3	0.43	0.60
ATR	4	3.4	2	3.5	8	5.8	1.0	0.39
BRCA1	3	2.5	0		7	5.1	-	0.34

**Table 5:** Prevalence of most common amplifications in ER-negative/HER2-negative claudin-low breast cancers, claudin-low breast cancers with ER and/or HER2 positivity and of basal breast cancers. Data are from METABRIC. Two statistical comparisons are presented for each gene (except for cases with no mutations), the first is between the two claudin-low groups and the second is between the two ER-negative/HER2-negative groups. The Fisher’s exact test has been used for all comparisons.

		Claudin-Low				Basal			
Chromosome Locus	Gene	ER-/HER2- (*n* = 130)	%	Non-ER-/HER2- (*n* = 60)	%	ER-/HER2- Basal (*n* = 143)	%	*p* for Comparison between the 2 Claudin-Low Groups	*p* for Comparison between the 2 ER-/HER2- Groups
8q24.21	MYC	26	20	12	20	61	42.7	1.0	0.0001
8q24.21	POU5F1B	27	20.8	11	18.3	60	42	0.84	0.0002
1q21.2	MCL1	12	9.2	0		35	24.5	0.01	0.001
1q23.1	NTRK1	14	10.8	2	3.3	35	24.5	0.1	0.004
11q13.3	CCND1	4	3.1	7	11.7	9	6.3	0.03	0.26
17q12	ERBB2	3	2.3	14	23.5	8	5.6	0.0001	0.22
8p11.23	NSD3	8	6.2	9	15	13	9.1	0.057	0.49

**Table 6:** Clinical and pathologic characteristics of ER-positive/HER2-negative/low proliferation/luminal A and ER-positive/HER2-negative/low proliferation/claudin-low breast cancers. Data are from METABRIC. The Fisher’s exact test has been used for all comparisons. NA: not available.

Characteristic	All ER+/HER2-/Low Proliferation (*n* = 640)	%	ER+/HER2-/Low Proliferation/Luminal A (*n* = 471)	%	ER+/HER2-/Low Proliferation/Claudin-Low (*n* = 39)	%	*p*
Menopause status							
Pre-	120	18.7	83	17.6	4	10.3	0.37
Post-	520	81.3	388	82.4	35	89.7	
Grade							
I/II	482	79.9	365	81.7	28	77.8	0.51
III	121	20.1	82	18.3	8	22.2	
NA	37		24		3		
Histology							
Ductal	412	65.3	311	66.7	26	66.7	0.37
Lobular	80	12.7	48	10.3	7	17.8	
Mixed	107	17	83	17.8	4	10.3	
Other	32	5	24	5.2	2	5.2	
NA	9		5		0		
Specimen cellularity							
Low	92	14.8	43	9.3	8	22.9	0.009
Intermediate	291	46.9	221	47.6	19	54.2	
High	238	38.3	200	43.1	8	22.9	
NA	19		7		4		

**Table 7:** TMB and integrative clusters in ER-positive/HER2-negative/low proliferation/luminal A and ER-positive/HER2-negative/low proliferation/claudin-low breast cancers. Data are from METABRIC. NA: not available. For the integrative clusters, comparisons for prevalent clusters 3, 4ER+, 7 and 8 in either group are presented. The Fisher’s exact test has been used for all comparisons.

Characteristic	All ER+/HER2-/Low Proliferation (*n* = 640)	%	ER+/HER2-/Low Proliferation/Luminal A (*n* = 471)	%	ER+/HER2-/Low Proliferation/Claudin-Low (*n* = 39)	%	*p*
TMB							
≤10 mutations/Mb	568	93.7	423	93.8	33	100	0.7
>10 mutations/Mb	38	6.3	28	6.2	0		
NA	34		20		6		
Integrative Cluster							
IntClust 1	10	1.6	4	0.8	1	2.6	
IntClust 2	15	2.3	11	2.3	0		
IntClust 3	203	31.7	164	34.8	5	12.8	0.004
IntClust 4ER+	143	22.3	72	15.3	29	74.4	0.0001
IntClust 4ER-	7	1.1	1	0.2	0		
IntClust 5	0		0		0		
IntClust 6	14	2.2	10	2.1	1	2.6	
IntClust 7	92	14.4	76	16.1	2	5.1	0.06
IntClust 8	149	23.3	128	27.2	0		0.0001
IntClust 9	7	1.1	5	1.1	1	2.6	
IntClust 10	0		0		0		

**Table 8:** Prevalence of most common mutations in ER-positive/HER2-negative/low proliferation/luminal A and ER-positive/HER2-negative/low proliferation/claudin-low breast cancers. Data are from METABRIC. The Fisher’s exact test has been used for all comparisons.

Gene	All ER+/HER2-/Low Proliferation (*n* = 619)	%	ER+/HER2-/Low Proliferation/Luminal A (*n* = 471)	%	ER+/HER2-/Low Proliferation/Claudin-Low (*n* = 39)	%	*p*
PIK3CA	377	60.9	290	63.6	16	42.1	0.02
TP53	62	10	35	7.7	6	15.8	0.11
KMT2C	96	15.5	83	18.2	2	5.3	0.04
GATA3	97	15.7	85	18.6	2	5.3	0.04
CDH1	80	12.9	62	13.6	3	7.9	0.45
MAP3K1	105	17	82	18	3	7.9	0.17
KMT2D	46	7.4	38	8.3	0		0.1
PDE4DIP	38	6.1	28	6.1	3	7.9	0.72
TBX3	48	7.8	35	7.7	3	7.9	1.0
NOTCH1	30	4.8	22	4.8	2	5.3	0.7
ARID1A	28	4.5	20	4.4	0		0.38
CBFB	55	8.9	42	9.2	4	10.5	0.76
NCOR2	28	4.5	23	5	2	5.3	1.0
AKT1	26	4.2	20	4.4	1	2.6	1.0
PTEN	22	3.6	14	3.1	4	10.5	0.04
RB1	6	1	5	1.1	0		1.0
ROS1	13	2.1	9	2	1	2.6	0.55
ATR	20	3.2	13	2.9	2	5.3	0.32
BRCA1	6	1	6	1.3	0		1.0

**Table 9:** Prevalence of most common amplifications in in ER-positive/HER2-negative/low proliferation/luminal A and ER-positive/HER2-negative/low proliferation/claudin-low breast cancers. Data are from METABRIC. The Fisher’s exact test has been used for all comparisons.

Chromosome Locus	Gene	All ER+/HER2-/Low Proliferation (*n* = 640)	%	ER+/HER2-/Low Proliferation/Luminal A (*n* = 471)	%	ER+/HER2-/Low Proliferation/Claudin-Low (*n* = 39)	%	*p*
8q24.21	MYC	54	8.4	35	7.4	6	15.4	0.11
8q24.21	POU5F1B	54	8.4	35	7.4	6	15.4	0.11
1q21.2	MCL1	136	21.3	114	24.2	0		0.0001
1q23.1	NTRK1	156	24.4	130	27.6	2	5.1	0.001
11q13.3	CCND1	59	9.2	37	7.9	5	12.8	0.35
17q12	ERBB2	15	2.3	10	2.1	2	5.1	0.23
8p11.23	NSD3	54	8.4	37	7.9	7	17.9	0.06

## Data Availability

There are no data available related to this article beyond the data presented within this article.

## References

[1] Perou C.M., Sørlie T., Eisen M.B., van de Rijn M., Jeffrey S.S., Rees C.A., Pollack J.R., Ross D.T., Johnsen H., Akslen L.A. (2000). Molecular portraits of human breast tumours. Nature.

[2] Sørlie T., Perou C.M., Tibshirani R., Aas T., Geisler S., Johnsen H., Hastie T., Eisen M.B., van de Rijn M., Jeffrey S.S. (2001). Gene expression patterns of breast carcinomas distinguish tumor subclasses with clinical implications. Proc. Natl. Acad. Sci. USA.

[3] Kensler K.H., Sankar V.N., Wang J., Zhang X., Rubadue C.A., Baker G.M., Parker J.S., Hoadley K.A., Stancu A.L., Pyle M.E. (2019). PAM50 Molecular Intrinsic Subtypes in the Nurses’ Health Study Cohorts. Cancer Epidemiol. Biomark. Prev..

[4] Prat A., Chaudhury A., Solovieff N., Paré L., Martinez D., Chic N., Martínez-Sáez O., Brasó-Maristany F., Lteif A., Taran T. (2021). Correlative Biomarker Analysis of Intrinsic Subtypes and Efficacy Across the MONALEESA Phase III Studies. J. Clin. Oncol..

[5] Prat A., Pineda E., Adamo B., Galván P., Fernández A., Gaba L., Díez M., Viladot M., Arance A., Muñoz M. (2015). Clinical implications of the intrinsic molecular subtypes of breast cancer. Breast.

[6] Schettini F., Brasó-Maristany F., Kuderer N.M., Prat A. (2022). A perspective on the development and lack of interchangeability of the breast cancer intrinsic subtypes. npj Breast Cancer.

[7] Herschkowitz J.I., Zhao W., Zhang M., Usary J., Murrow G., Edwards D., Knezevic J., Greene S.B., Darr D., Troester M.A. (2012). Comparative oncogenomics identifies breast tumors enriched in functional tumor-initiating cells. Proc. Natl. Acad. Sci. USA.

[8] Prat A., Perou C.M. (2011). Deconstructing the molecular portraits of breast cancer. Mol. Oncol..

[9] Lehmann B.D., Jovanović B., Chen X., Estrada M.V., Johnson K.N., Shyr Y., Moses H.L., Sanders M.E., Pietenpol J.A. (2016). Refinement of Triple-Negative Breast Cancer Molecular Subtypes: Implications for Neoadjuvant Chemotherapy Selection. PLoS ONE.

[10] Burstein M.D., Tsimelzon A., Poage G.M., Covington K.R., Contreras A., Fuqua S.A., Savage M.I., Osborne C.K., Hilsenbeck S.G., Chang J.C. (2015). Comprehensive genomic analysis identifies novel subtypes and targets of triple-negative breast cancer. Clin. Cancer Res..

[11] Jiang Y.Z., Ma D., Suo C., Shi J., Xue M., Hu X., Xiao Y., Yu K.D., Liu Y.R., Yu Y. (2019). Genomic and Transcriptomic Landscape of Triple-Negative Breast Cancers: Subtypes and Treatment Strategies. Cancer Cell.

[12] Asleh K., Riaz N., Nielsen T.O. (2022). Heterogeneity of triple negative breast cancer: Current advances in subtyping and treatment implications. J. Exp. Clin. Cancer Res..

[13] Lehmann B.D., Colaprico A., Silva T.C., Chen J., An H., Ban Y., Huang H., Wang L., James J.L., Balko J.M. (2021). Multi-omics analysis identifies therapeutic vulnerabilities in triple-negative breast cancer subtypes. Nat. Commun..

[14] Mertins P., Mani D.R., Ruggles K.V., Gillette M.A., Clauser K.R., Wang P., Wang X., Qiao J.W., Cao S., Petralia F. (2016). Proteogenomics connects somatic mutations to signalling in breast cancer. Nature.

[15] Fougner C., Bergholtz H., Norum J.H., Sørlie T. (2020). Re-definition of claudin-low as a breast cancer phenotype. Nat. Commun..

[16] Pereira B., Chin S.F., Rueda O.M., Vollan H.K., Provenzano E., Bardwell H.A., Pugh M., Jones L., Russell R., Sammut S.J. (2016). The somatic mutation profiles of 2433 breast cancers refines their genomic and transcriptomic landscapes. Nat. Commun..

[17] Gao J., Aksoy B.A., Dogrusoz U., Dresdner G., Gross B., Sumer S.O., Sun Y., Jacobsen A., Sinha R., Larsson E. (2013). Integrative analysis of complex cancer genomics and clinical profiles using the cBioPortal. Sci. Signal..

[18] Cerami E., Gao J., Dogrusoz U., Gross B.E., Sumer S.O., Aksoy B.A., Jacobsen A., Byrne C.J., Heuer M.L., Larsson E. (2012). The cBio Cancer Genomics Portal: An open platform for exploring multidimensional cancer genomics data. Cancer Discov..

[19] Russnes H.G., Lingjærde O.C., Børresen-Dale A.L., Caldas C. (2017). Breast Cancer Molecular Stratification: From Intrinsic Subtypes to Integrative Clusters. Am. J. Pathol..

[20] Danzinger S., Hielscher N., Izsó M., Metzler J., Trinkl C., Pfeifer C., Tendl-Schulz K., Singer C.F. (2021). Invasive lobular carcinoma: Clinicopathological features and subtypes. J. Int. Med. Res..

[21] Oliphant M.U.J., Kong D., Zhou H., Lewis M.T., Ford H.L. (2020). Two Sides of the Same Coin: The Role of Developmental pathways and pluripotency factors in normal mammary stem cells and breast cancer metastasis. J. Mammary Gland Biol. Neoplasia.

[22] Yang J., Antin P., Berx G., Blanpain C., Brabletz T., Bronner M., Campbell K., Cano A., Casanova J., Christofori G. (2020). Guidelines and definitions for research on epithelial-mesenchymal transition. Nat. Rev. Mol. Cell Biol..

[23] Brabletz T., Kalluri R., Nieto M.A., Weinberg R.A. (2018). EMT in cancer. Nat. Rev. Cancer.

[24] Voutsadakis I.A. (2012). The ubiquitin-proteasome system and signal transduction pathways regulating Epithelial Mesenchymal transition of cancer. J. Biomed. Sci..

[25] Kalluri R., Weinberg R.A. (2009). The basics of epithelial-mesenchymal transition. J. Clin. Investig..

[26] Voutsadakis I.A. (2012). Ubiquitination and the Ubiquitin-Proteasome System as regulators of transcription and transcription factors in epithelial mesenchymal transition of cancer. Tumour Biol..

[27] Voutsadakis I.A. (2019). HER2 in stemness and epithelial-mesenchymal plasticity of breast cancer. Clin. Transl. Oncol..

[28] Mani S.A., Guo W., Liao M.J., Eaton E.N., Ayyanan A., Zhou A.Y., Brooks M., Reinhard F., Zhang C.C., Shipitsin M. (2008). The epithelial-mesenchymal transition generates cells with properties of stem cells. Cell.

[29] Voutsadakis I.A. (2015). The network of pluripotency, epithelial-mesenchymal transition, and prognosis of breast cancer. Breast Cancer (Dove Med. Press).

[30] Hollier B.G., Tinnirello A.A., Werden S.J., Evans K.W., Taube J.H., Sarkar T.R., Sphyris N., Shariati M., Kumar S.V., Battula V.L. (2013). FOXC2 expression links epithelial-mesenchymal transition and stem cell properties in breast cancer. Cancer Res..

[31] Chimge N.O., Baniwal S.K., Little G.H., Chen Y.B., Kahn M., Tripathy D., Borok Z., Frenkel B. (2011). Regulation of breast cancer metastasis by Runx2 and estrogen signaling: The role of SNAI2. Breast Cancer Res..

[32] Voutsadakis I.A. (2016). Epithelial-Mesenchymal Transition (EMT) and Regulation of EMT Factors by Steroid Nuclear Receptors in Breast Cancer: A Review and in Silico Investigation. J. Clin. Med..

[33] Prat A., Parker J.S., Karginova O., Fan C., Livasy C., Herschkowitz J.I., He X., Perou C.M. (2010). Phenotypic and molecular characterization of the claudin-low intrinsic subtype of breast cancer. Breast Cancer Res..

[34] Da Cruz Paula A., Lopes C. (2017). Implications of Different Cancer Stem Cell Phenotypes in Breast Cancer. Anticancer Res..

[35] Dias K., Dvorkin-Gheva A., Hallett R.M., Wu Y., Hassell J., Pond G.R., Levine M., Whelan T., Bane A.L. (2017). Claudin-Low Breast Cancer; Clinical & Pathological Characteristics. PLoS ONE.

[36] Pommier R.M., Sanlaville A., Tonon L., Kielbassa J., Thomas E., Ferrari A., Sertier A.S., Hollande F., Martinez P., Tissier A. (2020). Comprehensive characterization of claudin-low breast tumors reflects the impact of the cell-of-origin on cancer evolution. Nat. Commun..

[37] Morel A.P., Ginestier C., Pommier R.M., Cabaud O., Ruiz E., Wicinski J., Devouassoux-Shisheboran M., Combaret V., Finetti P., Chassot C. (2017). A stemness-related ZEB1-MSRB3 axis governs cellular pliancy and breast cancer genome stability. Nat. Med..

[38] Tao L., Xiang D., Xie Y., Bronson R.T., Li Z. (2017). Induced p53 loss in mouse luminal cells causes clonal expansion and development of mammary tumours. Nat. Commun..

[39] Silwal-Pandit L., Vollan H.K., Chin S.F., Rueda O.M., McKinney S., Osako T., Quigley D.A., Kristensen V.N., Aparicio S., Børresen-Dale A.L. (2014). TP53 mutation spectrum in breast cancer is subtype specific and has distinct prognostic relevance. Clin. Cancer Res..

[40] Rädler P.D., Wehde B.L., Triplett A.A., Shrestha H., Shepherd J.H., Pfefferle A.D., Rui H., Cardiff R.D., Perou C.M., Wagner K.U. (2021). Highly metastatic claudin-low mammary cancers can originate from luminal epithelial cells. Nat. Commun..

[41] Giusti V., Ruzzi F., Landuzzi L., Ianzano M.L., Laranga R., Nironi E., Scalambra L., Nicoletti G., De Giovanni C., Olivero M. (2021). Evolution of HER2-positive mammary carcinoma: HER2 loss reveals claudin-low traits in cancer progression. Oncogenesis.

[42] Dhasarathy A., Phadke D., Mav D., Shah R.R., Wade P.A. (2011). The transcription factors Snail and Slug activate the transforming growth factor-beta signaling pathway in breast cancer. PLoS ONE.

[43] Simpson K., Conquer-van Heumen G., Watson K.L., Roth M., Martin C.J., Moorehead R.A. (2021). Re-expression of miR-200s in claudin-low mammary tumor cells alters cell shape and reduces proliferation and invasion potentially through modulating other miRNAs and SUZ12 regulated genes. Cancer Cell Int..

[44] Lüönd F., Sugiyama N., Bill R., Bornes L., Hager C., Tang F., Santacroce N., Beisel C., Ivanek R., Bürglin T. (2021). Distinct contributions of partial and full EMT to breast cancer malignancy. Dev. Cell.

[45] Meyer-Schaller N., Tiede S., Ivanek R., Diepenbruck M., Christofori G. (2020). A dual role of Irf1 in maintaining epithelial identity but also enabling EMT and metastasis formation of breast cancer cells. Oncogene.

[46] Creighton C.J., Li X., Landis M., Dixon J.M., Neumeister V.M., Sjolund A., Rimm D.L., Wong H., Rodriguez A., Herschkowitz J.I. (2009). Residual breast cancers after conventional therapy display mesenchymal as well as tumor-initiating features. Proc. Natl. Acad. Sci. USA.

